# RFE based chondroplasty in wrist arthroscopy indicates high risk for chrondocytes especially for the bipolar application

**DOI:** 10.1186/s12891-015-0460-2

**Published:** 2015-01-31

**Authors:** Michaela Huber, Christoph Eder, Markus Loibl, Arne Berner, Johannes Zellner, Richard Kujat, Michael Nerlich, Sebastian Gehmert

**Affiliations:** Department of Trauma Surgery, University Medical Center Regensburg, Franz-Josef-Strauss-Allee 11, 93053 Regensburg, Germany; Center for Medical Biotechnology, University of Regensburg, Regensburg, Germany; Department of Orthopedic Surgery, University Hospital Basel, Basel, Switzerland

**Keywords:** Temperature, Subchondral, Chondroplasty, RFE, Wrist, Arthroscopy

## Abstract

**Background:**

The application of radiofrequency energy (RFE) has become widespread for surgical performed chondroplasty especially due to the anticipated sealing effect, however the safety of this procedure in the wrist remains unclear. The purpose of this study was to investigate the subchondral temperature during radiofrequency energy (RFE) application simulating chondroplasty in an arthroscopic setting of the wrist.

**Methods:**

A chondroplasty of the lunate fossa was performed during an arthroscopy setting on 14 cadaver arms using monopolar or biopolar RFE. The temperature was recorded simultaneously from 7 predefined anatomical landmarks.

**Results:**

The mean temperature for both application modes did not exceed more than 30°C at all measured points, except for the lunate fossa. The highest subchondral measured peak temperature was 49.35°C (monopolar) and 69.21°C (bipolar) in the lunate fossa. In addition, the temperature decreased for both radiofrequency (RF) devices depending on the distance of the sensors to the RF-probe.

**Conclusion:**

It remains to be questionable how safe RFE can be used for chondroplasty in wrist arthroscopy under continuous irrigation and constant movement to obtain the desired sealing effect. However, the bipolar device should be applied with more caution since peak temperature in the lunate fossa almost reached 70°C even under continuous irrigation.

## Background

Articular cartilage damage progress without treatment in size and grade due to increased permeability which eventually leads to fibrillation, delamination, and swelling [1]. In addition, cartilage affected by degeneration show reduced capacity to absorb compressive load and subsequently provoke crepitus and pain owing to the destructed cartilage surface [2]. Over the last decade radiofrequency energy (RFE) has been applied for chondroplasty of partial-thickness chondral defects in large joints with promising results [3]. Chrondoplasty is used with the intent to terminate or at least to decelerate the degradation of the fibrillated articular cartilage by smoothing the roughened cartilage surface without the degradation of surrounding intact cartilage. Noteworthy, patient’s symptoms have been reported to improve by limiting the degradation of articular cartilage [3,4]. The effect of cartilage smoothing has been termed as “sealing effect” and consistently reported in various studies in which chondroplasty showed an improved morphologic and histological stabilization of damaged cartilage by RFE [5-7].

Turner et al. demonstrated in a sheep animal model for the first time, that bipolar radiofrequency energy (bRFE) might be superior to conventional mechanical shaving (MS) for the treatment of chondromalacic cartilage based on histological criteria. However, viability of chondrocytes have not been analyzed in this study [8]. Recently, Edwards et al. demonstrated in an equine patella model that RFE for cartilage surface smoothing is superior to mechanical debridement (MD) due to fewer chondrocyte destruction while maintaining thicker cartilage [9]. However, various studies have been reported that chondrocyte cell death starts at temperatures between 45°C to 55°C [10,11]. Owens et al. reported in a prospective randomized trial that treatment with bRFE was superior to MD at 12 and 24 month postoperatively [12]. In contrast, Barber et al. did not detect any difference between the groups treated with MD or MD + mRFE (monopolar RFE) after 12 month. However, both studies differ by the applied RFE mode limiting the conclusion that the bipolar RFE might be the best applied modality [3].

Nevertheless, RFE for chondroplasty has been proved to induce less permeability of the cartilage which might lead to a long-term stability [13,14]. Moreover, the inflammatory process in the affected joint appeared to be reduced associated with the decrease of inflammatory mediators [7,15]. However, to date only a limited number of clinical trials exist that investigated the effect of RFE for chondroplasty in knee surgery. The empirical evidence regarding the safety and long-lasting effect of RFE is still conflicting hence caution is advised when performing such procedure on cartilage [16].

We decided to mimic a chondroplasty in our previously established hand cadaver model since RFE is frequently performed in the wrist by hand surgeons [17,18]. In addition, most of the current research data of RFE originated from studies of large joints. Furthermore, anatomical structures in the wrist are closely spaced and cartilage layer is much thinner when compared to the joints of hip or knee [19]. Thus, experimental data obtained from studies with large joints (i.e. knee, hip) cannot serve as a reliable reference in terms of risk assessment for RFE procedure in wrist.

Based on current literature we hypothesized, that time depended temperature peak might exceed a level leading to injury of chondrocytes and even bone. Furthermore, we expected a difference between bRFE and mRFE application, due to the different energy distribution of the systems.

## Methods

Fourteen upper limbs were obtained from seven fresh cadavers, The cadavers were provided from our Anatomical Institute of the University of Regensburg. Due to the regulations from our ethical board no further requirement exept the written consent of their body cessation for research of the deceased humans prior to death were necessary. The upper limbs were obtained from seven fresh cadavers who gave written consent prior to death. The arms were stored at −20°C without any further fixation and were only thawed to room temperature prior to starting the experiment.

**Figure 1:**
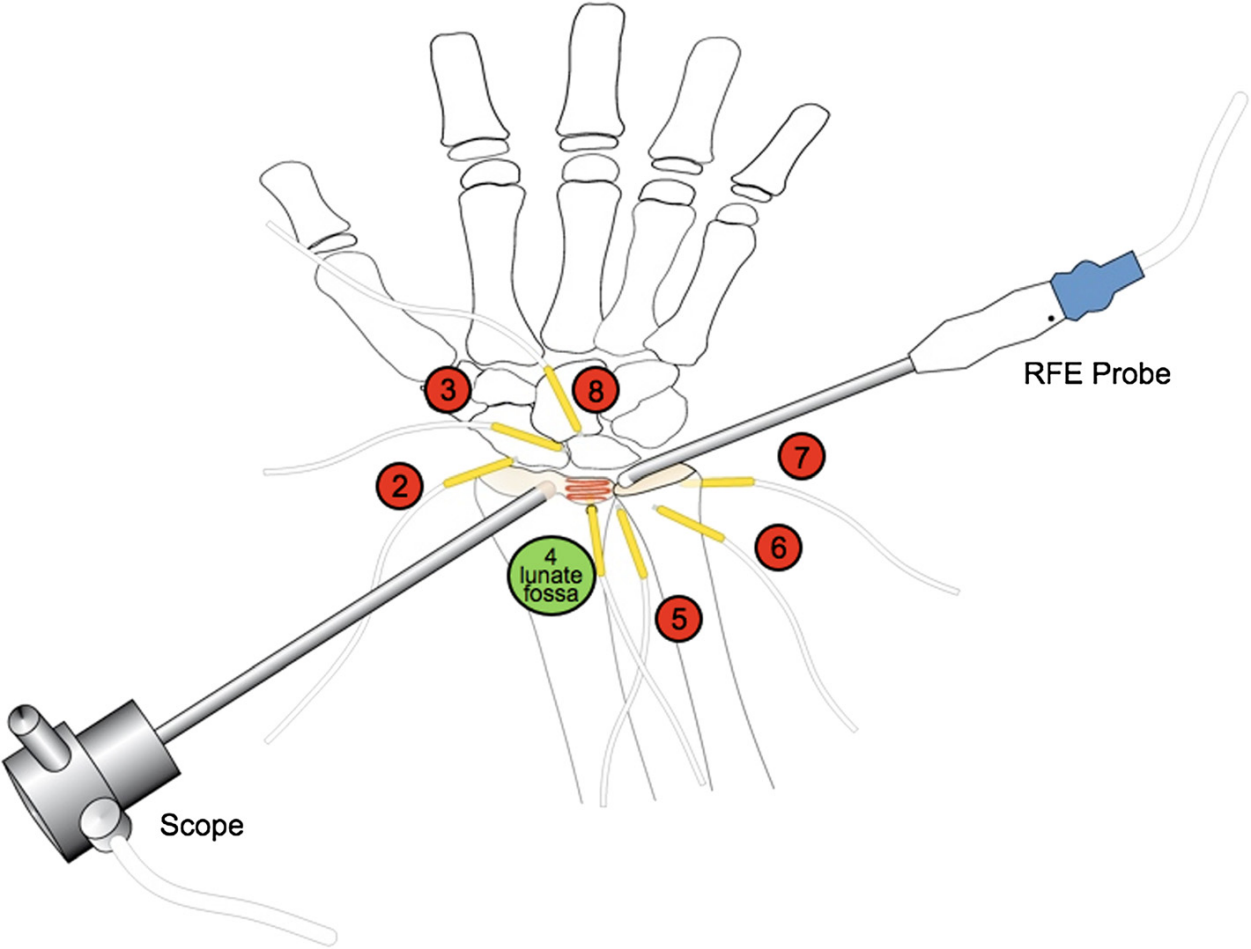
**Temperature sensing elements (TSE) were placed adjacent to sensible structures in the wrist in order to monitor temperature changes.** TSE 1 was used as a reference to detect the temperature of the irrigation fluid at room temperature of 20°C. Seven TSEs were implanted under direct visualization to: radial recess (rr), scapho-lunate ligament (sl), dorsal site and subchondral to the center of the lunate fossa (fl), intra-articularly at the distal radioulnar joint (druj), extra-articular tendon sheath of the 4/5 compartment (4/5), ulnar nerve in alignment to the extra-articular ulno-carpal joint (un), intra-articular at the center of the midcarpal joint (mc).

Temperature sensing elements (TSE) containing platinum-chip-sensors (Pt 1000, TYP PCA, 1.1505.10 M JUMO GmbH & Co.KG, Fulda, Germany) were used for all experiments, measuring two temperatures per second, with an accuracy of ± 0.1°C. Eight TSEs were used in total and placed adjacent to sensible structures in the wrist [20] in order to monitor temperature changes. TSE 1 was used as a reference to detect the temperature of the irrigation fluid at room temperature of 20°C. Six TSEs were implanted by a proper skin incision followed by blunt dissection through the capsule using 2.5x magnifying lens under direct visualization, to the following locations on the wrist: TSE 2 was inserted intra-articular into the radial recess (rr). TSE 3 was placed into the scapho-lunate ligament (sl). TSE 4 was positioned into a hole of 2 mm, which was drilled from the dorsal site and subchondral to the center of the lunate fossa (fl). TSE 5 was arranged intra-articularly at the distal radioulnar joint (druj) whereas TSE 6 was inserted into the extra-articular tendon sheath of the 4/5 compartment (4/5). TSE 7 was implanted adjacent to the ulnar nerve in alignment to the extra-articular ulno-carpal joint (un) (Figure 1). TSEs were fixed with a 4/0 Prolene suture and the capsule was closed.

Afterwards, the arms were fixed in the commercially available Acumed Arc Wrist Tower (Acumed, Hillsboro/OR, USA), where finger traps and maximum distraction was applied (Figure 1). The final TSE 8 was located intra-articular and fixed during arthroscopy at the center of the midcarpal joint (mc).

A standard wrist arthroscopy was performed using the 3–4 portal for the overview and 6R portal for the RF probe. VAPR II 2.3 mm side effect electrodes (Depuy Mitek, Westwood, MA, USA) were used in order to operate bipolar currency. A Monopolar OPES ablator for small joints (45° REF AR-9601SJ-45 OPES Ablator AR-9600; Arthrex, Naples, FL, USA) was applied for all monopolar procedures.

All wrists were initially flushed with NaCl 0.9% until a temperature of 20°C was reached by all probes. Irrigation was applied at a pressure of 50 mm Hg with an inflow rate of 50 mL/min. An 18-gauge needle in the 6U portal achieved the gravity-assisted outflow. Temperature was monitored and recorded by an 8-chanel custom build simultaneous measuring device.

The bipolar RF probe was used with a power of 60 W for ablation-mode and 45 W for coagulation-mode, whereas the monopolar RF probe settings were 20 W for cut-mode and 10 W for coagulation-mode.

The RF probe was continually moved backward and forward in a meandering pattern over the lunate fossa for a total of 45 s simulating a chondroplasty of the lunate fossa (Figure 1). In particular, RFE was first applied for 30s in the ablation-mode respectively the cut-mode followed without any break by 15 s coagulation-mode.

Afterwards, every wrist was dissected in order to validate correct placement of each TSE by measuring the distance to the location of the RF in millimeters.

Histograms and descriptive statistic were calculated to determine distributions and outliers. Values at p < 0.05 were considered as statistically significant. All statistical analysis was performed using the statistical software PASW Statistics 17 for Windows (SPSS Inc., Chicago, IL, USA). Since the underlying data were not normally distributed, non-parametric tests were applied (one-sided Spearman’s rho coefficient, Kendall’s tau coefficient and a two-tailed Mann–Whitney U test).

## Results

**Figure 2:**
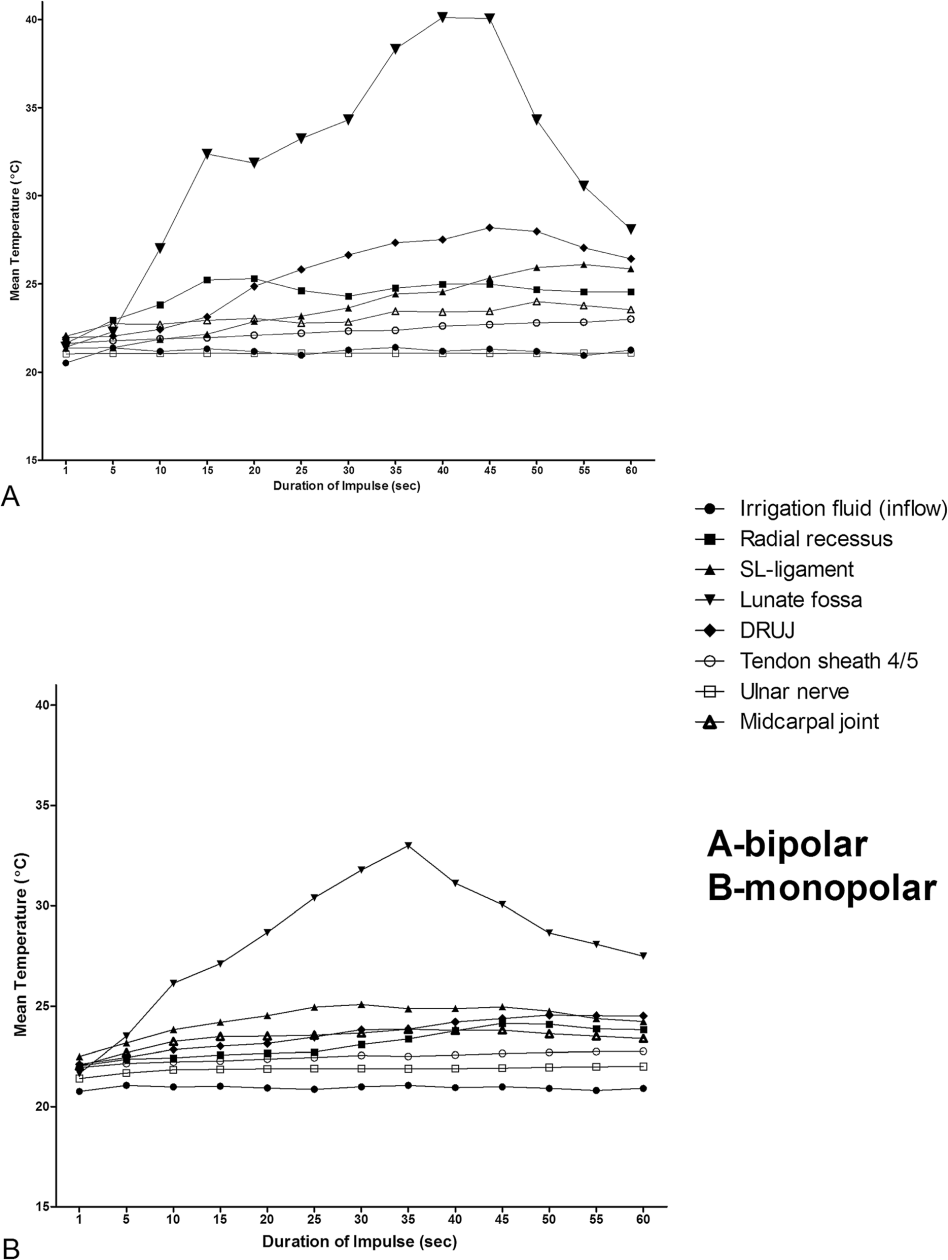
**Mean temperatures were recorded for all temperature sensing elements and displayed from 0 to 60 seconds for both RFE devices (A) bipolar, (B) monopolar.**

The application of bipolar RFE over 45 seconds significantly increased the mean temperature at all TSEs (p ≤ 0.002; Spearman’s rho coefficient > 0.4). Interestingly, the mean temperature during the bRFE application did not exceeded 30°C except for the lunate fossa. with a peak of 40.16 ± 11.42 C°. Noteworthy, after bRFE for 45 seconds a plateau-like curve was detected at the lunate fossa followed by a decrement under 30°C. In addition, the temperature increased even after switching to the coagulation-mode at the time point of 30 seconds (Figure 2A, Table 1).

**Table 1:** **Bipolar Device**

**Mean Temperature + SD (°C)**								
	**Irrigation fluid**	**Radial recessus**	**SL-ligament**	**Lunate fossa**	**DRUJ**	**Tendon sheath of 4/5 Comp**	**Ulnar nerve**	**Midcarpal joint**
0 s	20,52 ± 1,00	21,61 ± 1,48	21,35 ± 1,48	21,42 ± 0,93	21,94 ± 1,23	21,62 ± 0,68	21,04 ± 0,74	22,03 ± 1,05
5 s	21,02 ± 1,34	23,10 ± 1,89	21,46 ± 1,59	22,93 ± 1,04	22,23 ± 1,43	21,81 ± 0,65	21,07 ± 0,74	22,76 ± 1,50
10 s	21,02 ± 1,34	24,81 ± 5,25	21,95 ± 1,90	30,03 ± 12,63	22,55 ± 1,61	21,91 ± 0,69	21,07 ± 0,74	22,74 ± 1,37
15 s	21,24 ± 1,47	25,03 ± 3,36	22,11 ± 2,54	33,54 ± 14,30	24,08 ± 4,42	21,98 ± 0,75	21,08 ± 0,73	22,88 ± 1,52
20 s	21,31 ± 1,52	24,61 ± 2,75	22,90 ± 3,00	31,60 ± 7,16	24,91 ± 5,87	22,11 ± 0,83	21,08 ± 0,75	22,89 ± 1,48
30 s	20,54 ± 0,96	24,35 ± 2,32	23,80 ± 3,91	33,42 ± 8,49	27,10 ± 11,55	22,37 ± 1,22	21,08 ± 0,73	22,88 ± 1,15
40 s	21,03 ± 1,31	24,93 ± 2,97	24,64 ± 4,42	40,16 ± 11,42	27,72 ± 11,81	22,63 ± 2,13	21,07 ± 0,72	23,43 ± 1,93
45 s	21,23 ± 1,49	24,93 ± 3,15	25,52 ± 4,30	38,24 ± 9,36	28,40 ± 10,27	22,71 ± 2,26	21,07 ± 0,74	23,50 ± 2,20
60 s	21,45 ± 1,62	24,54 ± 2,00	25,61 ± 4,80	27,82 ± 3,55	26,29 ± 6,08	23,09 ± 3,00	21,10 ± 0,72	23,51 ± 1,70

As expected, the temperature decreased proportional to the distance of the RF-probe (p = 0.005; Kendall-Tau-b coefficient of correlation = 0.81).

Similar results were obtained for the monopolar system with a significant increase of the mean temperature at all measurement points (p ≤ 0.001; Spearman’s rho coefficient > 0.84). However, the maximum mean temperature only reached 33 ± 8.9 C° after 34 seconds in the lunate fossa followed by a decrease to 28°C. This is of interest, since switching the monopolar-device to the coagulation-mode after 30 s was associated with a decrease of the mean temperature, whereas the bipolar device was associated with a further temperature increase (Figure 2B, Table 2).

**Table 2:** **Monopolar Device**

**Mean Temperature + SD (°C)**								
	**Irrigation fluid**	**Radial recessus**	**SL-ligament**	**Lunate fossa**	**DRUJ**	**Tendon sheath of 4/5 Comp**	**Ulnar nerve**	**Midcarpal joint**
0 s	20,75 ± 1,45	21,85 ± 0,74	22,19 ± 1,02	21,64 ± 1,00	22,10 ± 0,56	21,78 ± 0,78	21,39 ± 1,28	22,02 ± 0,54
5 s	20,91 ± 1,53	22,35 ± 1,46	23,16 ± 1,51	24,07 ± 2,93	22,48 ± 1,07	22,13 ± 1,23	21,67 ± 1,74	22,92 ± 1,39
10 s	20,91 ± 1,53	22,41 ± 1,47	23,83 ± 1,66	25,88 ± 5,02	22,86 ± 1,44	22,20 ± 1,24	21,84 ± 2,03	23,27 ± 1,71
15 s	20,96 ± 1,59	22,56 ± 1,46	24,19 ± 2,05	26,99 ± 6,52	23,06 ± 1,52	22,24 ± 1,24	21,82 ± 2,04	23,54 ± 1,93
20 s	20,99 ± 1,65	22,65 ± 1,49	24,52 ± 2,58	29,02 ± 8,08	23,21 ± 1,58	22,34 ± 1,15	21,85 ± 2,03	23,49 ± 1,68
30 s	20,69 ± 1,34	23,09 ± 1,71	25,08 ± 3,19	31,66 ± 7,94	23,87 ± 2,26	22,52 ± 1,09	21,89 ± 2,02	23,65 ± 1,73
40 s	20,87 ± 1,42	23,77 ± 2,34	24,88 ± 3,11	30,91 ± 5,50	24,30 ± 3,09	22,54 ± 1,27	21,90 ± 2,04	23,80 ± 1,97
45 s	20,94 ± 1,46	24,15 ± 2,26	24,96 ± 2,96	29,71 ± 3,97	24,38 ± 3,13	22,62 ± 1,37	21,92 ± 2,04	23,83 ± 2,04
60 s	20,98 ± 1,50	23,82 ± 2,18	24,23 ± 2,33	27,42 ± 3,91	24,52 ± 3,20	22,75 ± 1,48	21,99 ± 2,08	23,38 ± 1,53

Similar to the bipolar system the temperature significantly decreased to the distance of the RF-probe (p = 0.002; Kendall-Tau-b coefficient of correlation = 0.905).

**Figure 3:**
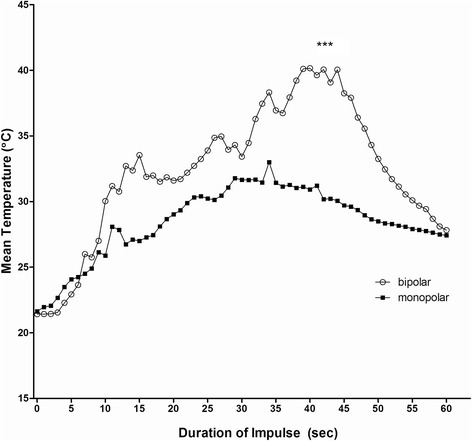
**Mean temperatures were recorded from the sensing element at the subchondral lunate fossa during bipolar and monoplar RFE application.** The mean temperature in the lunate fossa was significantly higher for the bipolar system when compared to the monopolar system (*** p < 0.001).

Comparison of mean temperature revealed significant higher temperature values in the radial recess, the lunate fossa and the DRUJ when using the bipolar system (p < 0.001). Interestingly, significantly higher temperatures were detected for the monoplar system at the sl-ligament, the 4/5 tendon sheath compartment, the ulnar nerve and the midcarpal joint (p ≤ 0.001). The mean temperature in the lunate fossa was significantly higher for the bipolar system when compared to the monopolar system (p < 0.001) (Figure 3).

**Figure 4:**
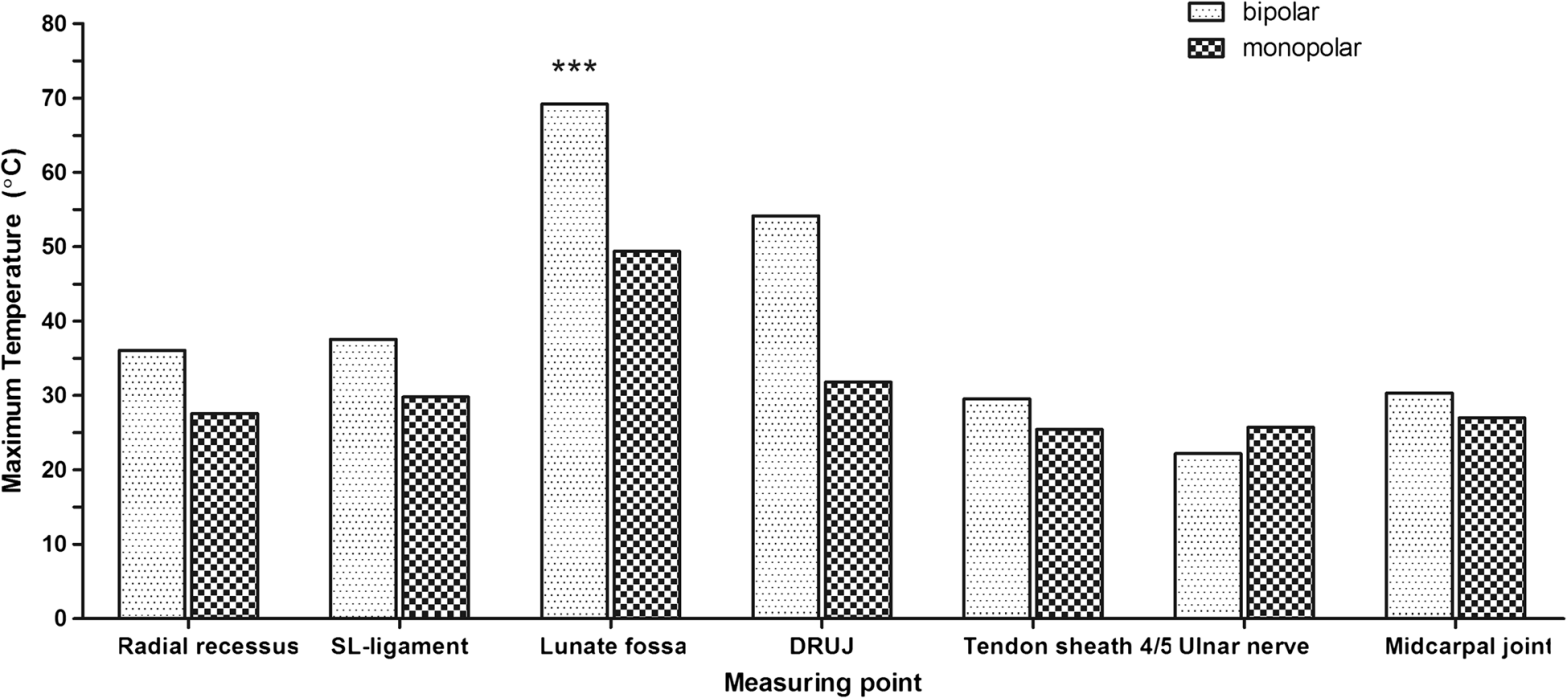
**The maximum temperature of 69.21°C was detected in the lunate fossa for the bipolar system whereas the peak temperature was only 49.35°C for the monoplar system at the same location.** In addition, peak temperature in the DRUJ reached 54.15°C when using the bipolar system but only 31.81°C for the monoploar application. The temperature of 50°C was never exceeded when using the monopolar system.

The maximum temperature was 69.21°C for the bipolar system detected in the lunate fossa whereas the peak temperature for the monoplar system in the lunate fossa was only 49.35°C. Application with the bipolar system induced temperatures over 50°C in 3 wrists in each at different time points (15 s, 30 s, 45 s) at the lunate fossa. In addition, when using the bipolar system a peak temperature of 54.15°C was detected in the DRUJ compared to 31.81°C for the monoploar System. The temperature of 50°C was never exceeded when using the monopolar system (Figure 4). The peak temperatures were significantly higher for the bipolar system (p =0.043) when compared to the monopolar RFE.

## Discussion

In this study we investigated the subchondral temperature profile during RFE application while mimicking a commonly performed chondroplasty in wrist arthroscopy. We found significantly higher subchondral mean temperatures and peak temperatures for the bipolar system when compared to the monopolar system. These results are in line with a previous study by Edwards et al. which measured cartilage matrix temperatures in different depth (200 μm, 500 μm, 2000 μm) during the application of RFE. The authors demonstrated chondrocytes death even at a depth of 2000 μm when using a bipolar system for 20s. In comparison application of mRFE induced an injury of chondrocytes only at a depth of 500 μm [21]. Hence, Edwards et al have raised concerns over RFE for chondroplasty especially when chondral matrix is less than 3 mm [22]. We consider these results important as normal thickness of cartilage layer in the wrist is around 0.7 to 1.2 mm [19] and cartilage damage can occur at temperature of 45°C to 55°C [10,11].

Lu et al. reported that the mold and seal effect on delaminated cartilage can be obtained in a time dependent manner whereas 15 s application of RFE is required in order to get a detectable effect. Noteworthy, a signicantly higher smoothing grade was present after 30s when compared to 20s applied. In the present study we decided to simulate a commonly used meander pattern for handling the RFE probe during the chondroplasty. In addition, a final coagulation mode was performed for 10s after a 30s ablation-mode or cut mode in order to achieve a potential smooth surface [23].

We detected subchondral peak temperatures of almost 70°C when using the bipolar system and 50°C for the monopolar system. These results underline the potential risk for cartilage not only for the bipolar but also for the monopolar system since cartilage damage can occur at temperature around 45°C. Interestingly, Lu et al. reported that bRFE caused significantly higher rate of chondrocyte death than mRFE in a bovine osteoarticular model. Moreover, bRFE was more associated with a full-thickness chondrocyte death including the subchondral bone [15].

Recent studies suggest a sufficient irrigation system [24,25] to reduce the temperature adjacent to the energy application. However, our data provide evidence that temperature levels remain critical for cartilage during chondroplasty especially when using the bipolar mode. Thus, it is highly questionable that the irrigation temperature has a cooling effect on the subchondral bone side.

Notably, our results indicate a different distribution of the heat when comparing bRFE and mRFE due to their different mode of operation [22,26]. Therefore, irrigation might have a cooling effect for the monopolar system since the currency runs towards the grounding electrode. We found that the temperature profile decreased for the monopolar device when switching to the lower energy setting whereas temperature values still increased for the bipolar system especially in the radial recess, the lunate fossa and the DRUJ. Peak temperature for the bRFE received 50°C after 15 s in 14.3% and after 24 s in 28.6% of the cases in both the lunate fossa and the DRUJ. However, this was not apparent for the monopolar device since the mean temperature did not exceed 30°C at these two specific locations. Our data indicate that even chondrocytes in the DRUJ might be under risk when using bRFE. In contrast, the mean temperature for the monopolar system was significantly higher in the sl ligament, the midcarpal joint, the 4/5 tendon sheath and the ulnar nerve.

But this might be caused by the location of the indifferent electrode which had to be placed at the posterior side of the forearm due to the experimental setting. It is well known that electrosurgical current during monopolar applications can cause tissue damage at patient’s skin. Especially, skin areas are affected which have contact to wet underground or cover while no appropriate indifferent electrode was attached to the patient. Fickeling et al. demonstrated that increasing current causes an increase of the temperature at the indifferent electrode since the dispersion of the current is limited by the size of the attached electrode [27]. Thus, the reported higher temperature in the sl ligament, the midcarpal joint, the 4/5 tendon sheath and the ulnar nerve during monopolar application might be disproportional high caused by small distance between monopolar probe and indifferent electrode.

Kosey et al. recommended a constant movement of the probe during RFE application in order to limit the risk of chondrocyte damage by minimizing the point heating [16]. However, our results indicate that even under probe movement a harmful temperature level can be reached at the side of treatment.

Noteworthy, chondroplasty exhibit a potential risk of osteonecrosis since Li et al. reported apoptosis and necrosis for osteoblasts in a temperature-range of 48-52°C [28]. In line, several cases of osteonecrosis have already been described after RFE in knee arthroscopy [29,30].

Our data support previous findings regarding peak temperatures in the subchondral bone for the bipolar system (70°C) and for the monopolar system (50°C) which underlines the potential risk for the subchondral bone. However, effects of RFE at the subchondral bone have been reported to be reversible [31]. The underlying repair mechanism might explain that no evidence of avascular necrosis (AVN) in MRI imaging was apparent 12 month after mRFE application along with shaver usage in knee arthroscopy [3].

Despite the potential risk of temperature injuries there is clinical evidence from prospective, randomized controlled trials, which shows an improved functional outcome and decreased pain after RFE application [16]. However, to date it remains unclear whether the pain reduction is caused by delayed cartilage degradation or coagulation of intraarticular sensory receptors. Thus, the application of RFE for chondroplasty still requires more research in order to estimate potential risk and real advantage [32]. Kosey reported a discrepancy between the estimated side effects of RFE and clinical outcomes. Moreover, he recommended further research focusing on the long-term effect of RFE and the probe design [16].

### Limitation

The experimental setup cannot be compared to an arthroscopy performed on patients due to the higher body temperature in living humans compared to the baseline cadaver temperature of 20°C. We assume hat the peak temperature might be up to 10°C to 13°C higher than measured results in our experiment since the body temperature in the extremities on living human is around 33°C to 35°C. We did not perform power analysis prior conducting this study because of similar data of recently published cadaver studies. In addition, the temperature of the employed irrigation fluid can alter the results.

## Conclusion

It remains debatable whether RFE is a safe application for chondroplasty in wrist arthroscopy under continuous irrigation and constant movement. However, the bipolar device should be applied with more caution since peak temperature in the lunate fossa almost reached 70°C even under continuous irrigation.

## Abbreviations

RFE: Radiofrequency energy
RF: Radiofrequency
bRFE: Bipolar radiofrequency energy
mRFE: Monopolar radiofrequency energy
MS: Mechanical shaving
MD: Mechanical debridement
°C: Degree Celsius
TSE: Temperature sensing elements
rr: Radial recess
sl: Scapholunate ligament
fl: Lunate fossa
druj: Distal radioulnar joint
4/5: Tendon sheath of the 4/5 compartment
un: Ulnar nerve
mc: Midcarpal joint
W: Watt
S: Seconds
